# Mucosal healing of ileum-mucosa-associated lymphoid tissue lymphoma after *Helicobacter pylori* eradication: a case report and literature review

**DOI:** 10.3389/fonc.2025.1544858

**Published:** 2025-08-21

**Authors:** Yihan Huang, Jiaying Jiang, Kui Jiang, Bangmao Wang, Tianyu Liu, Hailong Cao

**Affiliations:** Department of Gastroenterology and Hepatology, General Hospital, Tianjin Medical University, National Key Clinical Specialty, Tianjin Institute of Digestive Diseases, Tianjin Key Laboratory of Digestive Diseases, Tianjin, China

**Keywords:** mucosa-associated lymphoid tissue, lymphoma, ileum, *Helicobacter pylori* eradication, case report

## Abstract

Mucosa-associated lymphoid tissue (MALT) lymphoma of the small intestine is relatively rare, and the treatment guideline has not been established yet. Here we present a case of MALT lymphoma in the terminal ileum, which regressed after *Helicobacter pylori* (*H. pylori*) eradication. A 53-year-old man had complained of abdominal discomfort and underwent a gastrointestinal endoscopic examination. *H. pylori*-associated erosive gastritis was diagnosed, and superficial ulcerated lesions were also found in the terminal ileum. Histopathologic examination and immunohistochemical analyses of ileal biopsy specimens confirmed the diagnosis of MALT lymphoma. No distant lymph node metastasis or other organ involvement was detected in positron emission tomography/computed tomography. Surprisingly, the ileum reached mucosal healing after quadruple therapy regimens for *H. pylori* eradication without additional treatments. There were no signs of recurrence during the follow-up for 18 months. The unique case which located only in the ileum revealed that eradication of *H. pylori* might be an effective treatment and deserves further studies. Moreover, we also provide a detailed overview of recently published literature regarding the eradication treatment for intestinal MALT lymphoma.

## Introduction

Mucosa-associated lymphoid tissue (MALT) lymphoma was first introduced by Isaacson and Wright in 1983 (1), which usually appeared in organs lacking lymphoid tissues, and was thought to be caused by chronic antigen stimulation induced by persistent infection and/or autoimmune processes (2). The MALT lymphoma was classified as extranodal marginal zone B cell lymphoma by the World Health Organization and is a subtype of non-Hodgkin lymphomas, accounting for approximately 5%–8% of all B-cell lymphomas (3, 4). Most MALT lymphomas occur in the gastrointestinal (GI) tract, most commonly in the stomach (50%-60%), followed by the small intestine (30%), and large intestine (10%), and also in extra-gastrointestinal organs such as the salivary gland, thyroid gland, and skin (5, 6). Gastric MALT lymphoma is related with *Helicobacter pylori* (*H. pylori*) infection, and *H. pylori* eradication can successfully regress low-grade gastric MALT lymphoma (7–9). However, until now, the etiology and standard treatment strategy of small intestinal MALT lymphoma have not been established due to its rarity, and most cases were treated either with chemotherapy and/or surgically (10–13). In the present report, we describe a patient with MALT lymphoma in the terminal ileum that presented with erosive lesions. The lesions were fully regressed after quadruple therapy for *H. pylori* eradication. A detailed literature review for different treatments regarding ileum MALT lymphoma was also summarized.

## Case presentation

**Figure 1:**
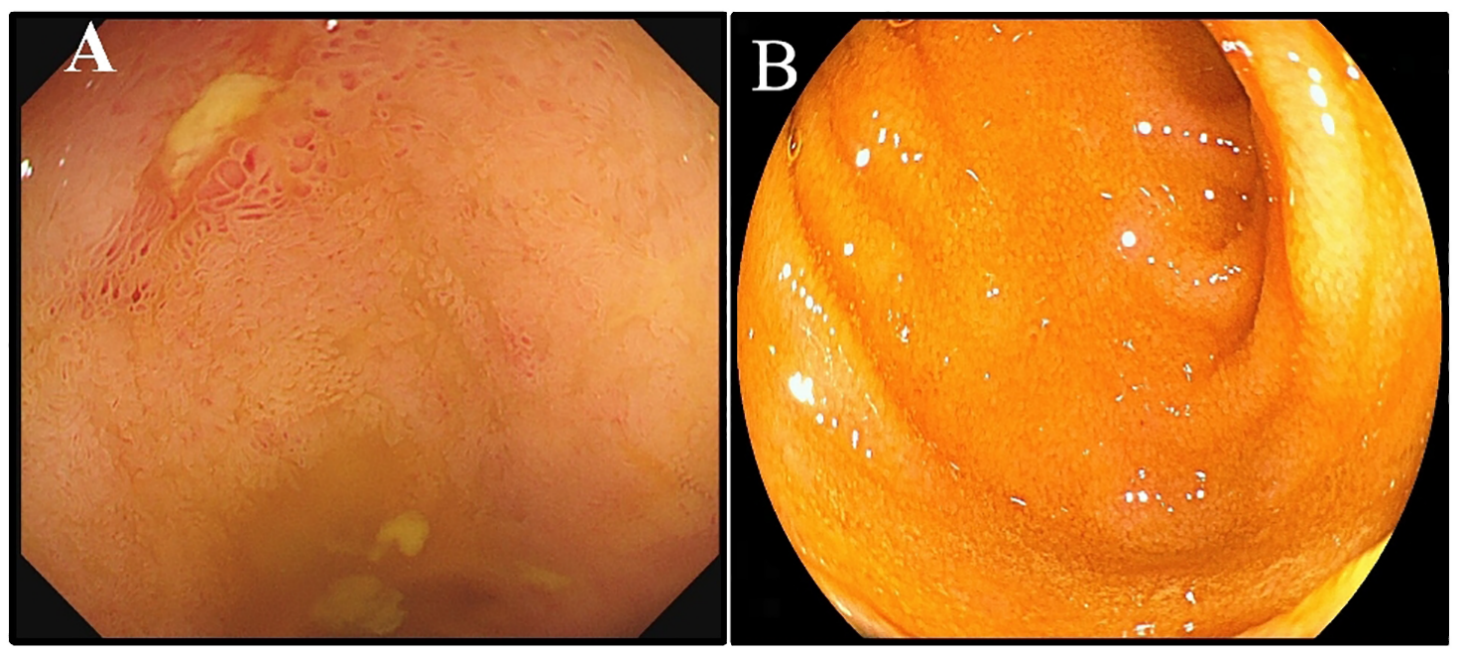
Endoscopic changes before and after treatment. **(A)** Colonoscopy performed due to multiple superficial ulcerated lesions in the terminal ileum. **(B)** The endoscopy result showed complete disappearance of the ileum MALT lymphoma.

**Figure 2:**
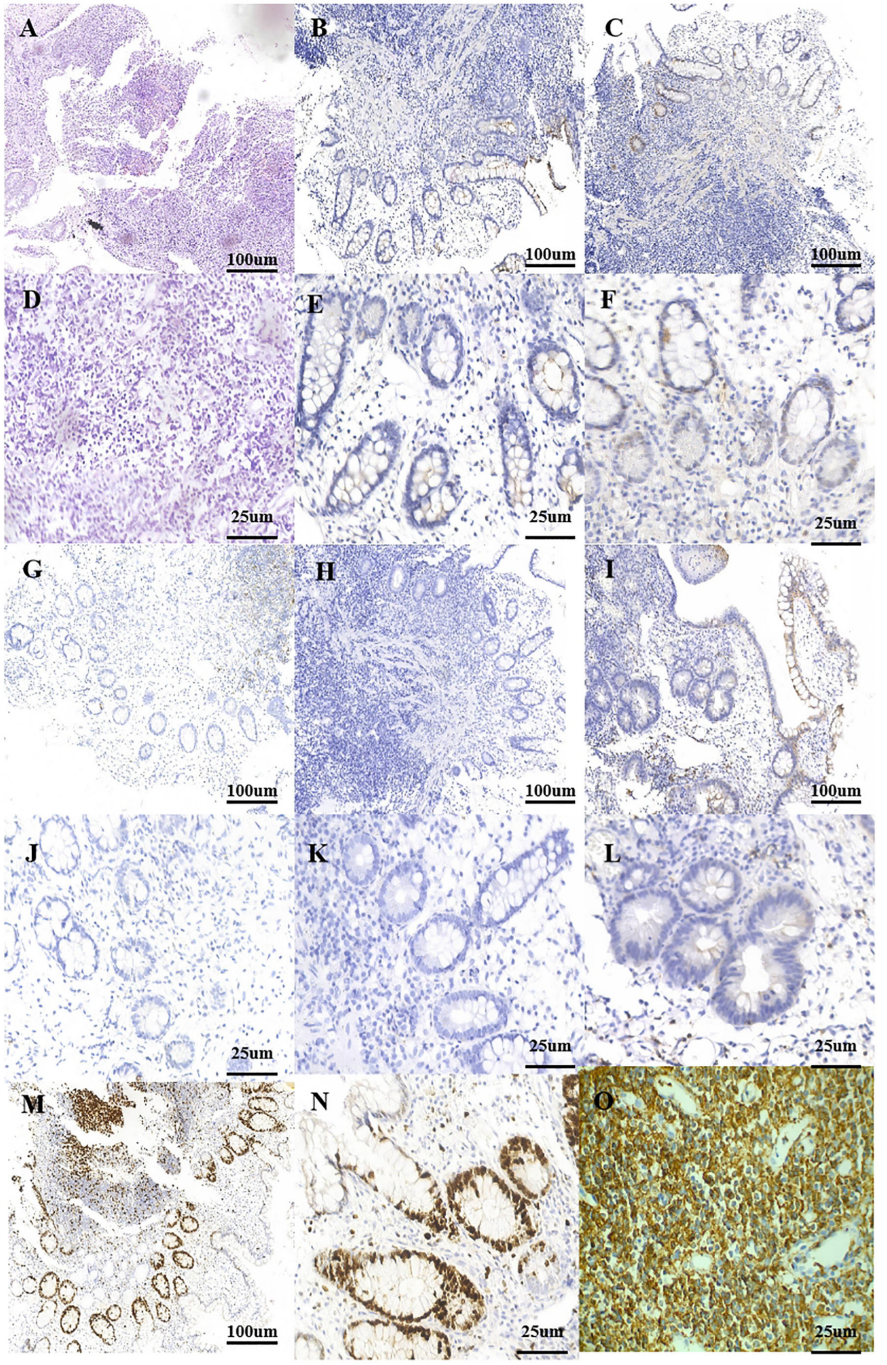
Postoperative pathology. **(A)** Small bowel with a dense and diffuse lymphocytic infiltrate (HE, ×10). **(B)** Typical cells are negative for the marker CD10 (IHE CD10, ×10). **(C)** Typical cells are negative for the marker cyclin D1 (IHE cyclin D1, ×10). **(D)** Small bowel with a dense and diffuse lymphocytic infiltrate (HE, ×40). **(E)** Typical cells are negative for the marker CD10 (IHE CD10, ×40). **(F)** Typical cells are negative for the marker cyclin D1 (IHE cyclin D1, ×40). **(G)** Typical cells are negative for the marker CD23 (IHE CD23, ×10). **(H)** Typical cells are negative for the marker BCL-6 (IHE BCL-6, ×10). **(I)** Typical cells are negative for the marker CD5 (IHE CD5, ×10). **(J)** Typical cells are negative for the marker CD23 (IHE CD23, ×40). **(K)** Typical cells are negative for the marker BCL-6 (IHE BCL-6, ×40). **(L)** Typical cells are negative for the marker CD5 (IHE CD5, ×40). **(M)** Ki-67 hot spot at about 10% (+) (IHE Ki-67, ×10). **(N)** Ki-67 hot spot at about 10% (+) (IHE Ki-67, ×40). **(O)** Typical cells are diffusely positive for the marker CD20 (IHE CD20, ×40). IHC, immunohistochemistry.

**Figure 3:**
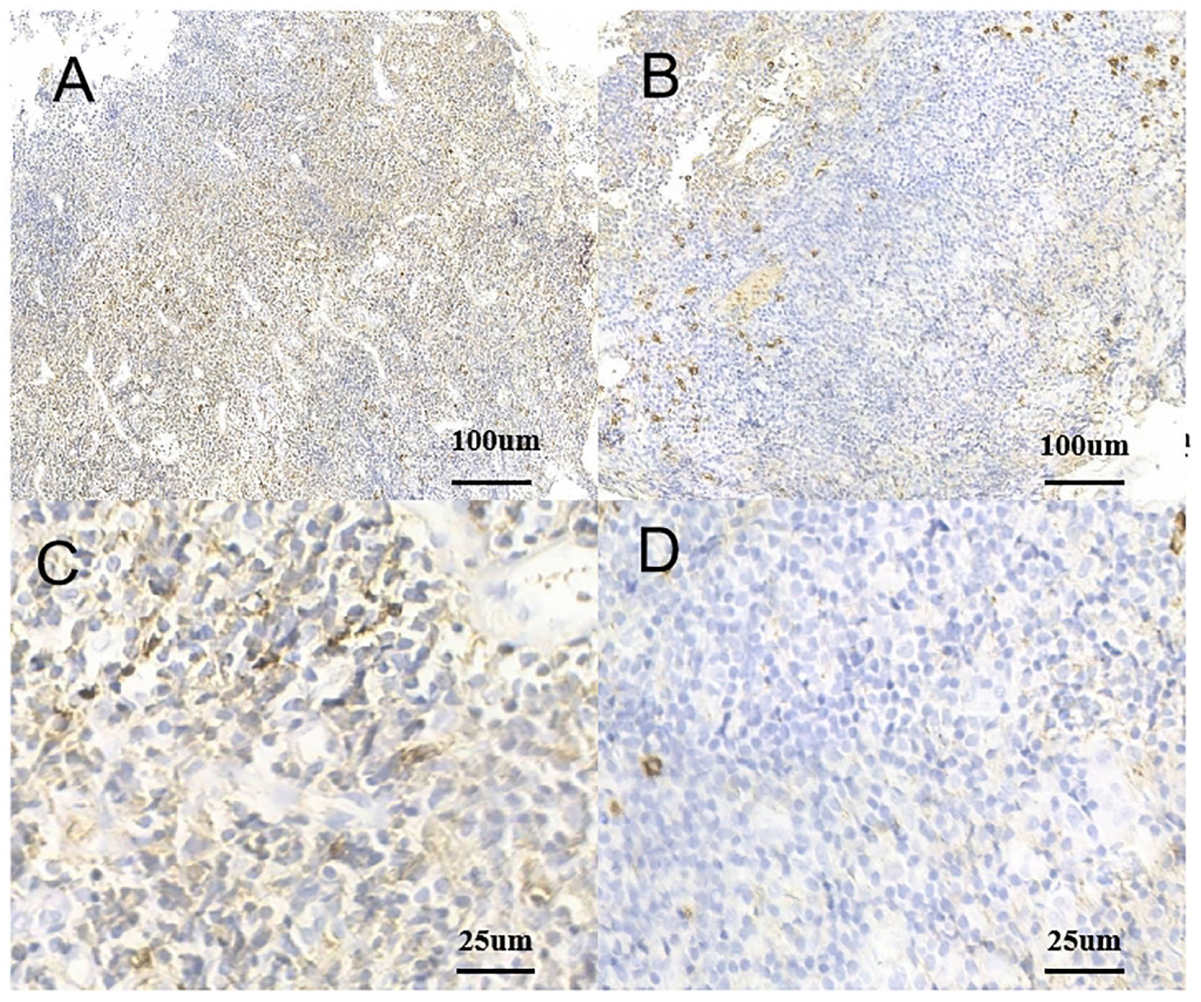
Monoclonality of tumor cells. **(A)** Positive rate of kappa light chain (IHC kappa, ×10). **(B)** Positive rate of lambda light chain (IHC lambda, ×40). **(C)** Positive rate of kappa light chain (IHC kappa, ×10). **(D)** Positive rate of lambda light chain (IHC lambda, ×40). IHC, immunohistochemistry.

**Figure 4:**
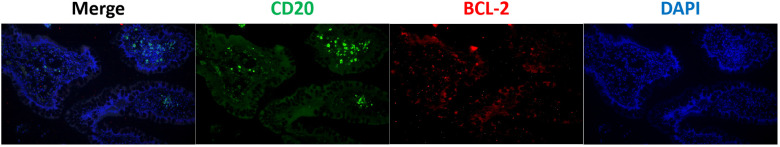
Co-expression of CD20 and BCL-2 by FISH strain. FISH, fluorescent *in situ* hybridization.

A 53-year-old man was admitted with complaints of abdominal discomfort without other symptoms, such as nausea, vomiting, diarrhea, constipation, and fever. He denied taking non-steroidal anti-inflammatory drugs. Upon gastroscopy, superficial erosive lesions in the antrum were found, and a histological evaluation of biopsy showed low-grade inflammation. The carbon-13 urea breath test for *H. pylori* infection was positive. Moreover, colonoscopy showed multiple superficial ulcerated lesions in the terminal ileum (**Figure 1A**). Hematoxylin–eosin staining revealed lymphoepithelial lesions with diffuse infiltration of atypical small lymphocytes in the lamina propria and muscularis mucosae (**Figures 2A, D**). Immunohistochemical staining showed diffuse positive for CD20 and Bcl-2 (**Figure 2O**) and negative for CD5, CD10, CD23, cyclin D1, and BCL-6 (**Figures 2B–L**). Ki-67 showed hot spot at about 10% (+) (**Figures 2M, N**). In addition, monoclonality was demonstrated by light chain restriction experiments (**Figure 3**). To rule out the possibility of a reactive lymphoid proliferation with co-expression of CD20 and BCL-2, we evaluated the co-expression of CD20 and BCL-2 by FISH strain (**Figure 4**). In addition, capsule endoscopy showed no lesions in other parts of the small intestine. The histopathological and phenotypic features were consistent with MALT lymphoma. Positron emission tomography/computed tomography showed no evidence of metastasis or any other abnormal lesions. According to the Lugano classification (12), the stage of this case was stage I. The patient received 14 days of *H. pylori* eradication therapy (esomeprazole, 20 mg bid; bismuth potassium citrate, 200 mg bid; amoxicillin, 1,000 mg bid; and clarithromycin, 500 mg bid) without any additional treatment. The carbon-13 urea breath test was repeated 4 weeks after treatment and confirmed successful eradication. At 3 months later, a repeated endoscopy and histological examination showed the complete disappearance of both gastric lesions and ileum MALT lymphoma (**Figure 1B**). The patient was recurrence-free at follow-up after 18 months.

## Discussion

Although MALT lymphoma may occur in multiple parts of the gastrointestinal tract, the stomach is the predominant localization. *H. pylori* infection is confirmed to be closely associated with gastric MALT lymphoma, and eradication of *H. pylori* is widely recognized as the first-line treatment for low-grade gastric MALT lymphoma (8, 14, 15). Previously, the findings of Gong, Eun Jeong et al. have also demonstrated the efficacy of *H. pylori* eradication as initial therapy for gastric MALT lymphoma (16). In contrast, for MALT lymphoma of the small intestine, the controversy continues on the current therapy regarding this entity. The cases with different treatments for ileum MALT lymphoma are summarized in **Supplementary Table 1** (11, 13, 17–28). In this case, the manifestation of mucosal healing of the terminal ileum was achieved after the successful eradication of *H. pylori*, and it could be considered a priority option.

MALT lymphomas arise from post-germinal center marginal zone B cells, which are positive for superficial Ig and pan B antigens CD19, CD20, CD79, and Bcl-2 and negative for CD5, CD10, CD23, cyclin D1, and Bcl-6 (29). In the present case, the immunohistochemical results were positive for CD20 and Bcl-2 and negative for CD10 and cyclin D1. Bacterial infection has been postulated to play a critical role in the pathogenesis of MALT lymphomas, such as *H. pylori*, *Borrelia afzelii*, and *Campylobacter jejuni* (30–33). Moreover, Du MQ et al. (34) reported that the intestinal lesions could be secondary to *H. pylori-*induced gastric MALT lymphoma. This suggested that antigen stimulation may play a critical role in the clonal expansion of low-grade MALT lymphomas. The present case was positive for *H. pylori* infection; however, MALT lymphoma was found only in the terminal ileum, but not in the stomach. The regression of the lesion was achieved after the successful eradication of *H. pylori*, and regular follow-up has, so far, not detected any evidence of recurrence. Thus, we speculated that the MALT lymphoma in our case was secondary to some infection, most likely *H. pylori*. Although controversy exists with regard to the antibiotic treatment for extra-gastric MALT lymphoma, some reports have described the effective remission of intestinal MALT lymphoma by eradication therapy in *H. pylori*-positive patients, even in negative cases (2, 23, 35–47) (**Supplementary Table 2**). These suggested that *H. pylori* or unknown antibiotic-sensitive microorganisms may play a critical role in the development of intestinal MALT lymphoma.

Notably, while our case and some literature highlight successful mucosal healing post-eradication, other reports emphasize that ileal MALT lymphoma may not always respond to *H. pylori*-targeted therapy, underscoring the need for individualized management—for instance, Ohashi et al. (48) described a rare case of terminal ileum MALT lymphoma without *Helicobacter pylori* infection in a patient; thus, antibiotic treatment targeting *H. pylori* might be ineffective in this scenario. Similarly, Hirata et al. (12) reported a case of small intestine MALT lymphoma presenting as multiple lymphoma polyps, where the lymphoma persisted despite the successful eradication of *H. pylori*. These examples illustrate that variations in treatment outcomes for anti-*Helicobacter* therapy can arise due to differences in disease location, lymphoma type, or whether *H. pylori* is the primary driving factor. These contrasting outcomes highlight the importance of comprehensive diagnostic workups to identify patients unlikely to benefit from antibiotic therapy alone. Further studies are needed to clarify the predictors of treatment response in ileal MALT lymphoma.

*C. jejuni* is increasingly recognized as a pathogen associated with immunoproliferative disorders, particularly mucosa-associated lymphoid tissue (MALT) lymphoma of the small intestine. Chronic infection with *C. jejuni* can induce persistent antigenic stimulation and chronic inflammation, fostering a microenvironment conducive to lymphoid hyperplasia and eventual malignant transformation. Studies such as those by Lecuit et al. (49) and Isaacson et al. (50) have identified *C. jejuni* in small intestinal MALT lymphoma tissues, with molecular evidence linking bacterial presence to clonal B-cell proliferation. Antibiotic eradication of *C. jejuni* has shown promising therapeutic potential in early-stage disease, akin to *H. pylori* eradication in gastric MALT lymphoma—for instance, regimens combining macrolides (e.g., clarithromycin) or fluoroquinolones (e.g., ciprofloxacin) with proton pump inhibitors have led to complete histological remission in some patients. This underscores the importance of identifying and targeting bacterial triggers, as antibiotic therapy may obviate the need for aggressive interventions in susceptible cases (12). However, treatment efficacy depends on early diagnosis, highlighting the clinical relevance of routine *C. jejuni* screening in suspected small intestinal lymphoproliferative disorders. Regrettably, our study was unable to test for *C. jejuni* due to technical constraints. Specifically, the detection of *C. jejuni* requires specialized methodologies, such as microaerobic bacterial culture, species-specific PCR, or fluorescence *in situ* hybridization (FISH), which were unavailable in our laboratory during the study period. Additionally, archival tissue samples lacked sufficiently fresh material for optimal molecular analysis, further limiting our capacity to assess bacterial presence. We acknowledge this as a critical limitation, as undetected *C. jejuni* infection could influence disease progression or treatment responsiveness in subsets of patients. Future studies incorporating multiplex PCR, metagenomic sequencing, or serological assays would help address this gap.

## Conclusion

We reported a rare case of MALT lymphoma in the terminal ileum, which completely resolved after *H. pylori* eradication. The eradication of treatable infectious organisms seems to be the first-step treatment option in patients with ileum MALT lymphoma, especially for *H. pylori*-positive patients. Large-scale clinical trials in multiple centers are required to investigate the etiology of ileum MALT lymphomas and establish a standard treatment strategy.

## Data Availability

The original contributions presented in the study are included in the article/**Supplementary Material**. Further inquiries can be directed to the corresponding authors.
