# Supplementary material for: Mucosal healing of ileum-mucosa-associated lymphoid tissue lymphoma after Helicobacter pylori eradication: a case report and literature review
Source: Front Oncol. 2025 Aug 21;15:1544858. doi: 10.3389/fonc.2025.1544858 (PMC12408310; doi:10.3389/fonc.2025.1544858)
Supplement: Supplementary file 1 [file DataSheet1.pdf]

Supplementary Material

Supplementary Tables

Table 1. Summary of ileum MALT lymphoma cases treated by different methods

First author (ref. no.)	Country	Publication year	Age (years)/ Sex	*Hp*	Treatment	Response	Follow-up (months)	Relapse
Kikuchi Y(24)	Japan	2005	80/female	—	Eradication*	CR	NR	NR
Makino Y(26)	Japan	2010	38/male	—	Spontaneous Regression	CR	15	No
Terada T(13)	Japan	2013	34/female	—	chemotherapy	NR	NR	NR
Dhull AK(11)	India	2014	55/male	NR	Surgery+chemo therapy	CR	12	No
Kinkade Z(25)	USA	2015	58/male	NR	Surgery+chemo therapy	CR	6	NR
Srinivasan AP(28)	India	2015	22/male	NR	Surgery	NR	NR	NR
Rosat A(27)	Spain	2016	45/female	NR	Surgery+chemo therapy	CR	24	No
Adams KM(17))		2016	83/female	NR	Surgery+chemo therapy	NR	NR	NR
Kawasaki K(23)	Japan	2019	77/male	NR	Surgery+chemo therapy	NR	NR	NR
Bennani A(20)	Morocco	2019	50/male	NR	Surgery+chemo therapy	CR	10	No

Álvarez-Nava Torrego MT(18)	España	2019	46/male	－	NR	NR	NR	NR
de Figueiredo VLP(22)	Brazil	2022	57/female	NR	chemotherapy	CR	NR	NR
Badwaik N(19)	India	2022	38/male	NR	Surgery	CR	6	No
Da B(21)	China	2024	32/male	NR	Surgery+chemo therapy	CR	NR	NR

CR: complete response; NR: not reported

*amoxicillin＋clarithromycin

Table 2. Summary of cases of effective remission of intestinal MALT lymphoma by antibiotics treatment

First author (ref. no.)	Country	Publication year	Age (years)/ Sex	*Hp*	Location	Scheme	Response	Follow-up (months)	Relapse
Matsumoto T(41)	Japan	1997	72/female	＋	Rectum	OAC	CR	3	No
Inoue F(40)	Japan	1999	62/female	－	Rectum	L_2AC	CR	12	No
Raderer M(2)	Austria	2000	67/male	＋	Colon	CMO	CR	36	No

Nakamura S(43)	Japan	2001	65/female	＋	Duodenal	RACM	CR	NR	NR
			48/female	＋	Duodenal	RAC	PR	NR	NR
Nakase H(44)	Japan	2002	66/female	－	Colon and Rectum	L_2ATM	CR	18	No
			33/female	－	Rectum	AC	CR	10	No
			62/female	－	Rectum	OAC	CR	6	No
Hisabe T(38)	Japan	2002	70/female	－	Rectum	L_1	CR	20	No
Hori K(39)	Japan	2004	83/female	＋	Rectum	G	CR	7	No
Dohden K(37)	Japan	2004	60/female	＋	Rectum	L_1	CR	NR	NR
Kikuchi Y(24)	Japan	2005	71/male	－	Rectum	AC	CR	12	No
			80/female	－	Ileum, Cecum and Rectum	AC	CR: Ileum and Cecum<br>PR: Rectum	NR	NR
			70/female	－	Rectum	AC	CR	20	No
Ahlawat S(35)	USA	2005	57/female	－	Rectum	OAC	CR	12	No
Niino D(46)	Japan	2010	67/female	＋	Rectum	AC	CR	5	No
			68/female	＋	Rectum	AC	CR	5	No
			76/female	NR	Rectum	AC	CR	5	No
			80/female	＋	Rectum	AC	CR	5	No

			46/female	NR	Rectum	AC	CR	5	No
De Sanctis V(36)	Italy	2012	66/male	+	Rectum	L_2AC	CR	34	No
Ohara E(47)	Japan	2012	53/male	−	Rectum	RAC	CR	36	No
Matsumoto T(42)	Japan	2013	80/female	+	Colon	L_1	CR	6	No
Nam MJ(45)	Korea	2017	54/female	−	Cecum and Rectum	OAC	CR	21	No

CR: complete response; PR: partial response; NR: not reported.

A amoxicillin, C clarithromycin, G gatifloxacin, L_1 levofloxacin, L_2 lansoprazole, M metronidazole, O omeprazole, R rabeprazole, T tetracycline
